# Extraction Optimization of Mucilage from Seeds of *Mimosa pudica* by Response Surface Methodology

**DOI:** 10.3390/polym14091904

**Published:** 2022-05-06

**Authors:** Syed Nasir Abbas Bukhari, Arshad Ali, Muhammad Ajaz Hussain, Muhammad Tayyab, Nasser F. Alotaibi, Mervat A. Elsherif, Kashaf Junaid, Hasan Ejaz

**Affiliations:** 1 Department of Pharmaceutical Chemistry, College of Pharmacy, Jouf University, Sakaka 72388, Saudi Arabia; 2 Institute of Chemistry, University of Sargodha, Sargodha 40100, Pakistan; arshadali04@yahoo.com; 3 Department of Pharmacy, Quaid-i-Azam University, Islamabad 45320, Pakistan; ph.tayyab@gmail.com; 4 Chemistry Department, College of Science, Jouf University, Sakaka 72388, Saudi Arabia; nfalotaibi@ju.edu.sa (N.F.A.); maelsherif@ju.edu.sa (M.A.E.); 5 Department of Clinical Laboratory Sciences, College of Applied Medical Sciences, Jouf University, Sakaka 72388, Saudi Arabia; kjunaid@ju.edu.sa (K.J.); hetariq@ju.edu.sa (H.E.)

**Keywords:** *Mimosa pudica* mucilage, extraction optimization, Box-Behnken design, response surface methodology, pH-responsive on–off switching, zero-order release

## Abstract

*Mimosa pudica* seed mucilage (MPM) is composed of glucuronoxylan, which is a swellable, pH-responsive and non-toxic biomaterial. Herein, we aimed to extract MPM from *M. pudica* seeds (MP seeds) to ascertain optimization of extraction conditions to get highest yield by response surface methodology, via Box-Behnken design (RSM-BBD). MPM was extracted from MP seeds by a hot water extraction method. The effects of four different parameters on the extraction yield of MPM were evaluated: pH of the extraction medium (1–10), seed/water contact time (1–12 h), the temperature of extraction medium (30–90 °C), and seed/water ratio (1:5–1:35 *w*/*v*). The maximum yield of MPM obtained by Design-Expert software was 10.66% (10.66 g/100 g) at pH 7, seed/water contact time of 6 h, extraction temperature of 50 °C, and seed/water ratio of 1:20 *w*/*v*. The *p* values of ANOVA were found to be less than 0.0001, which indicated that the extraction yield of MPM was significantly affected by all the study parameters. The results revealed that pH and extraction temperature were the most significant factors affecting the yield of MPM. MPM in compressed tablet form showed pH-responsive on–off switching behavior at pH 7.4 and 1.2 in a reversible manner. MPM in compressed tablet form sustained the release of itopride for 16 h following a super case-II transport mechanism and zero-order release kinetics.

## 1. Introduction

Mucilage from plant seeds has been extensively used in food systems as additives, emulsifiers, stabilizers, gelling agents, and texture modifiers, due to the safety profile, ease of availability, low cost, and biodegradable nature of mucilage [1,2,3,4]. Mucilaginous materials are polysaccharides in nature and have been extensively used in every sphere of life [5,6,7,8,9].

*Mimosa pudica* (Family, *Mimosaceae*) is a native Brazilian plant. Its height is 1 to 2 cm. The plant as a whole is enriched with phytochemicals. Its seeds (MP seeds) are brown and 2.5 mm in length. MP seeds have been utilized for the treatment of ulcers, piles, constipation, snake bites, depression, and smallpox [10]. MP seeds release mucilage (MPM) upon soaking in water. MPM contains glucuronoxylan, which is composed of d-xylose and d-glucuronic acid [11,12]. The glucuronoxylan from MP seeds is a water-swellable, pH-responsive, bio-compatible, non-toxic biomaterial [13,14]. MP seeds are commercially available, and many studies have been reported on their utilization as pharmaceutical and industrial excipients. However, no study is available in the literature so far for the optimization of extraction conditions at which maximum yield of MPM can be obtained to further broaden its spectrum of applications.

The optimization of the mucilage extraction yield by a single extraction factor is a costly and time-consuming process. Moreover, where there are multiple independent factors involved in the extraction process of mucilage from plant seeds, the quadratic and interaction terms of independent factors also affect the mucilage yield [15]. Therefore, due to the increase in research activities on plant seed mucilage, their extraction needs design of experiment (DOE) to optimize the ideal extraction conditions and to save time and cost. Response surface methodology (RSM) is a collection of mathematical and statistical tools for designing experiments to get ideal extraction conditions and elucidation of the combined effect of independent factors on extraction yield [16]. Many researchers have effectively used RSM for optimizing extraction conditions to get the maximum yield of mucilage, i.e., polysaccharides, from different parts of plants [17,18,19,20,21,22].

Herein, we aimed to extract mucilage from MP seeds and optimize the extraction conditions using RSM-BBD. The effects of four independent factors, i.e., pH of the extraction medium (A: 1–10), seed/water contact time (B: 1–12 h), extraction temperature (C: 30–90 °C), and seed/water ratio (D: 1:5 to 1:35 *w*/*v*) on the extraction yield of MPM were evaluated. The combined effect of the studied parameters, in terms of linear, quadratic, and interaction effects on the extraction yield of MPM, were also evaluated. Furthermore, we can report that MPM is a pH-responsive biomaterial for successful zero-order release of the standard drug itopride.

## 2. Materials and Methods

### 2.1. Materials

MP seeds were procured from the local market of District Sargodha, Pakistan. MP seeds were first cleaned manually and then sieved to eradicate any unwanted material and stored in an air-tight jar. Analytic grade reagents and solvents were purchased from Sigma-Aldrich Co., (St. Louis, MO, USA) and used as such, without further purification, to perform experiments. Buffers of pH 1.2, 6.8, and 7.4 were prepared according to the protocol given in the United States Pharmacopeia (USP 34-NP 29). Itopride was used as a standard drug and received from Dyson Research Laboratories, Lahore, as a gift sample. Deionized water (DI) was used for washing and preparation of necessary solutions and dilutions.

### 2.2. Methods

#### 2.2.1. Extraction of Mucilage

The mucilage (MPM) was extracted from MP seeds using the hot water extraction method. MP seeds were soaked in DI at 50 °C and allowed to swell for 6.0 h. The mucilage, i.e., the MPM released from the seed coats of swollen MP seeds, was isolated by using a clean cloth, and washed with *n*-hexane to remove non-polar/lipophilic substances. The MPM was further washed with ethanol to ensure it was free from polar impurities. Later, it was dried in a vacuum oven at 50 °C for 24 h. The completely dried MPM was ground and powdered by passing through a sieve no. 60, and stored in a vacuum desiccator.

#### 2.2.2. Calculation of Yield

The actual yield of MPM in percentage was calculated using Equation (1) [23].

(1) $$Extraction\;yield\;of\;\mathrm{MPM}\;\left(\%\right)=\frac{weight\;of\;extracted\;\mathrm{MPM}\;after\;drying}{weight\;of\;\mathrm{MP}\;seeds\;taken\;for\;extraction\;of\;\mathrm{MPM}}\times 100$$

#### 2.2.3. Experimental Design and Statistical Analysis

By following the aforesaid extraction procedure, the effect of four different parameters on the extraction yield of MPM were studied: pH of the extraction medium (*A*: 1–10), seed/water contact time (*B*: 1–12 h), the temperature of the extraction medium (*C*: 30–90 °C), and seed/water ratio (*D*: 1:5–1:35 *w*/*v*). These studies provided the effect of a single parameter on the extraction yield of MPM and were considered preliminarily experimental studies. The responses (% yield) obtained from these preliminary studies were further utilized for designing the model, according to RSM-BBD.

Three different levels were selected from the responses of individual parameters and were assigned as low (−), moderate (0), and high (+). For every individual parameter, the levels were: pH (low = 6, moderate = 7, high = 8), seed/water contact time (low = 4 h, moderate = 6 h, high = 8 h), extraction temperature (low = 30 °C, moderate = 50 °C, high = 70 °C), and seed/water ratio (low = 1:10 *w*/*v*, moderate = 1:20 *w*/*v*, high = 1:30 *w*/*v*). RSM-BBD was applied to the extracted yield data using the statistical package, Design-Expert version 12.0.3.0 (Stat-Ease Inc., Minneapolis, MN, USA). RSM-BBD provided the statistical treatment of the tested parameters on the yield of MPM in terms of regression (analysis of variance, ANOVA) and graphically (two dimensional, i.e., 2D response surface and three dimensional, i.e., 3D contours plots) analyses.

The ANOVA offers linear, quadratic, and composite interactions, through which their effects on the yield of MPM were also studied by noting *p*-values. The *F*-values and coefficients of regression, including (*R*^2^), adjusted-*R*^2^, and predicted-*R*^2^, were noted to check the significance of ANOVA. Also, the fitness level of the RSM-BBD model to the extraction yield data was predicted in terms of sum square error (SSE), standard error (DE), mean, coefficient of variance (% CV), adequate precision (ADP), and lack of fit.

The 3D response surface and 2D contour plots were acquired from RSM-BBD to obtain the points of optimum yield and optimum conditions. Also, from these plots, the combined effect of studied parameters, co-relationships between the parameters, and model desirability were studied. Moreover, the extraction yield data of MPM was further put into a second-order polynomial equation to assess the statistical significance of the RSM-BBD. A general linearized form of a second-order polynomial Equation (2) is presented below.

Y = α_o_ + α_1_A + α_2_B + α_3_C + α_4_D + α_11_A^2^ + α_22_B^2^ + α_33_C^2^ + α_44_D^2^ + α_1_α_2_AB + α_1_α_3_AC + α_1_α_4_AD + α_2_α_3_BC + α_2_α_4_BD + α_3_α_4_CD + E (2)

where, Y is the yield response (%). The A, B, C, and D represent the pH of the extraction medium, seed/water contact time, the temperature of the extraction medium, and seed/water ratio, respectively. The α_o_ is the intercept. α_1_, α_2_, α_3_, and α_4_ are the coefficient of linearity. α_11_, α_22_, α_33_, and α_44_ are the quadratic coefficients. α_1_α_2_, α_1_α_3_, α_1_α_4_, α_2_α_3_, α_2_α_4_, and α_3_α_4_ are the coefficient of interactions. E is the error function of the model.

#### 2.2.4. Evaluation of MPM as a Sustained Release Material

##### Preparation of MPM-Based Tablets

Itopride was used as a model drug to assess the potential of MPM as a pH-responsive and sustained release material. Three different oral tablet formulations (F1, F2, and F3), based on MPM and itopride, were prepared using the wet granulation method [13]. The composition of the tablets is given in Table 1. The mixture of MPM and itopride was prepared in a pestle and mortar and homogenized using a 5% solution of polyvinyl pyrrolidone (PVP) prepared in isopropyl alcohol. The mixture was sieved, granulated, dried in an oven at 50 °C under vacuum, lubricated using 5 mg of magnesium stearate, and pressed on a rotary press having a 9 mm flat surface punch. The hardness of every tablet was maintained between 6–9 kg/cm^2^.

##### pH-Responsive On–Off Switching Studies

The pH-responsive on–off switching (swelling and deswelling) properties of the oral tablet formulation F3 (having high concentration of MPM) were studied by alternatively immersing the tablet in beakers having 100 mL of each buffer of pH 7.4 (swelling medium) and pH 1.2 (deswelling medium), after enclosing the tablet in a pre-weighed tea bag [13]. The tea bag was removed from the swollen medium after pre-defined intervals of time and the swelling and deswelling responses were recorded using Equation (3). These experiments were conducted to record three cycles of swelling and deswelling studies. The swelling and deswelling experiments were performed three times and the mean of the values calculated as below.

(3) $$Swelling\;capacity\;\left(g/g\right)=\frac{W_{s}-W_{o}-W_{e}}{W_{o}}$$

where, *W_s_*, *W_o_*, and *W_e_* represent the weight of a wet tea bag having swollen tablet of MPM formulation F3, the weight of the dry tablet of MPM formulation F3, and the weight of an empty wet tea bag, respectively.

##### In Vitro Release Study of Itopride

The in vitro release of itopride from oral tablet formulation F3 was studied in a buffer of pH 6.8 (900 mL) for 16 h on USP Dissolution Apparatus II. The dissolution apparatus was run by maintaining the temperature of the dissolution medium at 37 ± 0.5 °C and the rotation speed of the paddles at 50 rpm. The sample (10 mL) was taken out from the dissolution medium after a pre-defined interval of time, filtered using a 0.45 μm nylon filter, necessarily diluted, and run on a UV-Vis spectrophotometer (UV-1600 Shimadzu, Germany) at 258 nm to record the absorbance. The dissolution medium was restored by adding the same amount of freshly prepared buffer of pH 6.8. The release study was also performed at pH 1.2 for 2 h and at pH 6.8 for the next 14 h to study the release behavior that mimics the pH and transit time of the gastrointestinal tract.

##### Drug Release Kinetics and Mechanism

The zero-order kinetic model (Equation (4)) [24] was used to study the rate of itopride release from F3 and the Korsmeyer-Peppas model (Equation (5)) [25] was applied to the release data. The mechanism of itopride release from F3 was evaluated.

(4) $$Q_{t}=K_{0}$$

where, *Q_t_* and *K*_0_ shows quantity of itopride release at time *t* and rate constant for zero-order kinetic model, respectively.

(5) $$\frac{M_{t}}{M_{\infty }}=k_{p}t^{n}$$

where, *M_t_*/*M*_∞_, *k_p_*, and *n* represent the quantity of itopride released at any time *t*, rate constant for the Korsmeyer-Peppas model, and diffusion exponent, respectively. The value of *n* describes the mechanism of drug release from oral tablet formulation. The value of *n* can be ≤0.45 for Fickian diffusion, from 0.45-0.89 for non-Fickian diffusion, =0.89 for case-II transport, and >0.89 for super case-II transport [26,27].

## 3. Results and Discussion

### 3.1. Extraction of Mucilage

The MPM was extracted from MP seeds by the hot water extraction method. MP seeds were soaked in DI. Water penetrated the microscopic pores of the MP seeds because of the swellable nature of glucuronoxylan present in MPM. MPM appeared as a colorless powdery material in dry form. Its yield was optimized by RSM-BBD.

### 3.2. Preliminary Studies for the Extraction Optimization of MPM Yield

#### 3.2.1. Effect of pH

**Figure 1:**
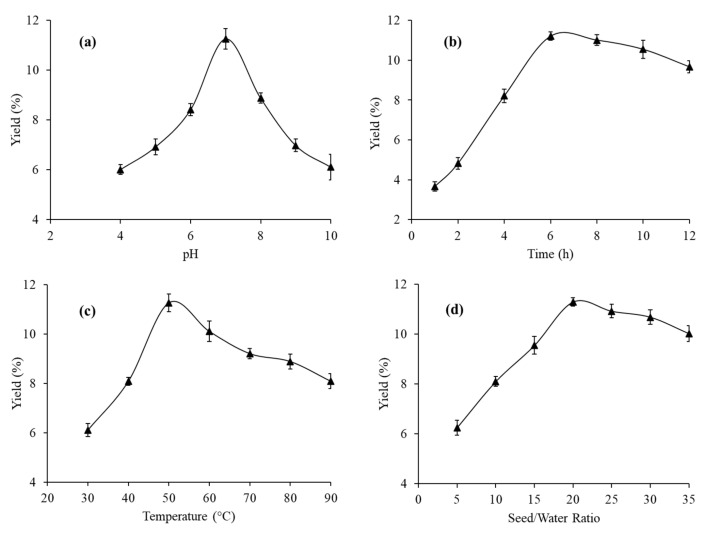
Effect of the pH of extraction medium (**a**), seed/water contact time (**b**), the temperature of the extraction medium (**c**), and seed/water ratio (**d**) on the yield of MPM extracted from MP seeds.

The selectivity of any material for pharmaceutical applications, particularly regarding drug delivery, is based on its pH-responsive nature. Therefore, it is essential to know the effect of pH on the extraction yield of the material. The effect of pH was evaluated by changing the pH of the extraction medium from 1 to 10. The rest of the extraction conditions, such as the seed/water contact time (6 h), extraction temperature (50 °C), and seed/water ratio (1:20 *w*/*v*), were kept constant. A negligible, i.e., less than 0.5%, yield of MPM was found at the lower pH ranging from 1–4 (not reported here). However, beyond pH 4.0 the yield of MPM increased and reached a maximum of 11.26% (9.0 g/100 g) at pH 7 = DI. On further increase in the pH, the yield of MPM dropped to 6.11% at pH 10.0. Moreover, it was noted that the yield of MPM was higher at an alkaline pH than that of an acidic pH (Figure 1a). This may be due to the fact that in an alkaline medium, due to possible hydrolysis, some of the insoluble polysaccharide fractions get converted into soluble ones [28]. A similar trend has previously been reported by Somboonpanyakul et al., Balke and Diosady, and Esteves et al. [29,30,31]. Hence, MPM extracted from MP seeds is a pH-responsive polysaccharide and could be an ideal candidate for pharmaceutical applications.

#### 3.2.2. Effect of Seed/Water Contact Time

To study the effect of seed/water contact time on the yield of MPM, this contact time was varied from 1–12 h. The other extraction conditions were adjusted at their optimum levels. It was revealed that as seed/water contact time increased from 1 to 6 h, the yield of MPM also increased abruptly, and after 6 h the yield of MPM decreased by a small extent (Figure 1b). As far as the extraction medium and seeds are in contact with each other, the exposure of water to seed increases, due to which more and more water enters into the pores of the seeds and acts as a driving force to remove mucilage from seeds [15,32]. Reports on the mucilage extraction from *Malva sylvestris* [29], cress seeds [28], and *Arctic chlorella* sp., [33] also favor greater yield in percentage at a longer extraction time.

#### 3.2.3. Effect of Temperature

The effect of extraction temperature on the yield of MPM was studied within temperatures ranging from 30 to 90 °C. The conditions of the pH 7, seed/water contact time of 6 h, and seed/water ratio 1:20 *w*/*v* were maintained. It was evaluated that initially at low temperature, i.e., at 30 °C, the yield of MPM was low and equal to 6.11%. However, with increase in temperature from 30 to 50 °C, the yield of MPM also increased from 6.11 to 11.27%. After 50 °C, the yield of MPM decreased to 8.09% at 90 °C (Figure 1c). As the maximum yield of MPM was achieved at 50 °C, this temperature was considered to be the optimum extraction temperature. The reason underlying this trend is that with increase in the temperature of the extraction medium the polysaccharide gets solubilized in it, due to which the value of the diffusion coefficient of the polysaccharide increases. At a high value of diffusion coefficient, a greater mass of polysaccharides is extracted from the plant seeds and this leads to an increase in the yield [34]. Another possible reason for this trend is that at high extraction temperatures, the seeds become less sticky and, consequently, release a high amount of mucilage. However, after the optimum temperature, the polysaccharide may degrade and release less mucilage [2]. Many other studies have reported a decrease in mucilage yield on increasing temperature of the extraction medium. which may be due to thermal degradation of the polysaccharides in the mucilage at high temperature [35]. Similar findings have been demonstrated for extraction optimization of chia seed mucilage [1] and *Alyssum homolocarpum* seed mucilage [36].

#### 3.2.4. Effect of Seed/Water Ratio

At pH 7, extraction temperature 50 °C, and contact time 6 h, the influence of seed/water ratio on the yield of MPM was studied by varying seed/water ratio from 1:5 to 1:35 *w*/*v*. The results obtained are incorporated in Figure 1d. On increasing seed/water ratio from 1:5 to 1:20, the yield of MPM also increased. At 1:20 seed/water ratio the maximum yield of MPM, i.e., 11.29% was achieved, and after that, no more significant increase or decrease in the yield was observed, due to the attainment of a state of dynamic equilibrium. At a high seed/water ratio, the water molecule of the extraction medium exerts more driving force on the seeds and pushes the mucilage to come out from the seeds [37,38]. Hence, the highest yield of MPM was achieved at seed/water ratio 1:20 *w*/*v*. The yield of mucilage extracted from *Dioscorea nipponica* [39] and *Phoenix dactylifera* [40] also reported similar results.

Conclusively, the above discussion shows that all the tested parameters have a significant effect on the yield of MPM extracted from MP seeds. The maximum yield, i.e., 11.29% (11.29 g/100 g), of MPM was obtained at pH 7.0, contact time 6 h, extraction temperature 50 °C, and seed/water ratio 1:20 *w*/*v*. Therefore, based on these preliminary experiments, three different levels, such as low (−), moderate (0), and high (+) for each parameter were selected and RSM-BBD containing 29 different sets of experimental runs were constructed (Table 2). At every set of experimental runs, MPM was extracted and its yield in percentage was tabulated and validated statistically by applying ANOVA.

### 3.3. Fitting of Model

Findings of 29 different experimental runs, according to RSM-BBD, are incorporated in Table 2, which includes the design, actual, and predicted yields of MPM. Both the actual and predicted yields were found very close to each other. The actual yield of MPM was found in the range of 3.68 to 11.25%. The yield data of MPM was fitted to the second-order polynomial response surface model and the following quadratic Equation (6) was obtained.

Actual yield (%) of MPM = 10.66 + 0.8858A + 0.7092B + 0.7992C + 0.61587D − 2.31A^2^ − 1.578B^2^ − 2.03C^2^ − 1.09D^2^ − 0.5925AB − 0.9700AC − 1.32AD + 0.4950BC + 0.4800BD + 1.64CD (6)

Table 3 shows the results of ANOVA regarding the RSM-BBD. As the *p*-value of ANOVA is significant, i.e., <0.001, it means that the RSM-BBD model is satisfactorily applied to the yield data for optimization of extraction conditions. The *p*-values in the case of linear independent parameters were found to be significant, i.e., *p* < 0.001, which shows that the yield of MPM was purely dependent on them. Among all the independent parameters, the pH of the extraction medium has a pronounced effect on the yield of MPM followed by extraction temperature, seed/water contact time, and seed/water ratio. Moreover, *p* < 0.0001 also indicated that the extraction yield of MPM related linearly to the studied parameters.

The yield of MPM was also found to be dependent on quadratic (A^2^, B^2^, C^2^, D^2^) and interaction (AB, AC, AD, BC, BD, and CD) terms. All the quadratic terms have a significant effect on the yield of MPM in the sequence of A^2^ > C^2^ > B^2^ > D^2^ by considering *p*-values. The interaction between pH vs. extraction temperature (AC), pH vs. seed/water ratio (AD), and extraction temperature vs. seed/water ratio (CD) was found highly significant because the *p*-values in these cases are less than 0.01. The interaction terms pH vs. contact time (AB), contact time vs. extraction temperature (BC), and contact time vs. seed/water ratio (BD) were found non-significant because the *p*-values in these cases are greater than 0.05. The effect of interaction terms on the yield of MPM was found in the order of CD > AD > AC > AB > BC > BD.

The adequacy of the RSM-BBD model for yield data was further assessed by comparing the values of *R*^2^, adjusted-*R*^2^, and predicted-*R*^2^. The difference between *R*^2^ (0.9425) and adjusted-*R*^2^ (0.8850) was found to be 0.0575 and the difference between *R*^2^ (0.9425) and predicted-*R*^2^ (0.7055) was found to be 0.237. This minute difference, and high value of *R*^2^, indicated that RSM-BBD is an adequate model for yield data of MPM [41].

The % CV describes the variation in the mean value and interprets the adequacy of the model for the studied response with great accuracy, high precision, and reliability of experiment. If the value of % CV is less than 10%, then the variation in the mean value is low and hence the response model will develop with great satisfaction and vice versa. In this research, the % CV value was found to be 8.92%. This low value of % CV indicated that the RSM-BBD model was adequate and desirable to identify the optimum extraction conditions for the highest yield of MPM from MP seeds. Moreover, a low % CV value is testament to the fact that the extraction experiments had high precision and reliability [42].

The value of ADP helps in predicting the signal-to-noise ratio as well as model desirability. Generally, the greater the value of ADP, the lower will be the signal-to-noise ratio and the higher will be the model desirability. The normal value of ADP is 4.0. This means that if the ADP for any designed model is greater than 4.0, then it is desirable for optimizing the extraction conditions. The AD*p* value was found to be 14.0462 which is greater than a normal value [43]. Hence, RSM-BBD is a desirable model for optimizing the yield of MPM.

Lack of fit is another vital factor to determine whether the RSM-BBD model successfully fits, or fails to fit, in representing the extraction yield data in the experimental domain. A significant lack of fit (*p* < 0.05) means that the model is not satisfactorily applied to the yield data and the corresponding response factors should not be included in the regression. Whereas, a non-significant lack of fit (*p* > 0.05) means that the model is satisfactorily applied to the yield data and the corresponding response factors should be included in the regression. This study found a non-significant lack of fit, i.e., 0.0760, for all independent factors at a 95% confidence interval. This means that all models can equally predict the corresponding responses accurately [38].

Therefore, it can be concluded that the second-order quadratic was the most significant model to identify the optimum conditions for the extraction of MPM from MP seeds.

### 3.4. Interpretation of Response Surface Plots

The 3D response surface and 2D contour plots were obtained from RSM-BBD to evaluate the combined effect of all the studied parameters on the extraction yield of MPM.

**Figure 2:**
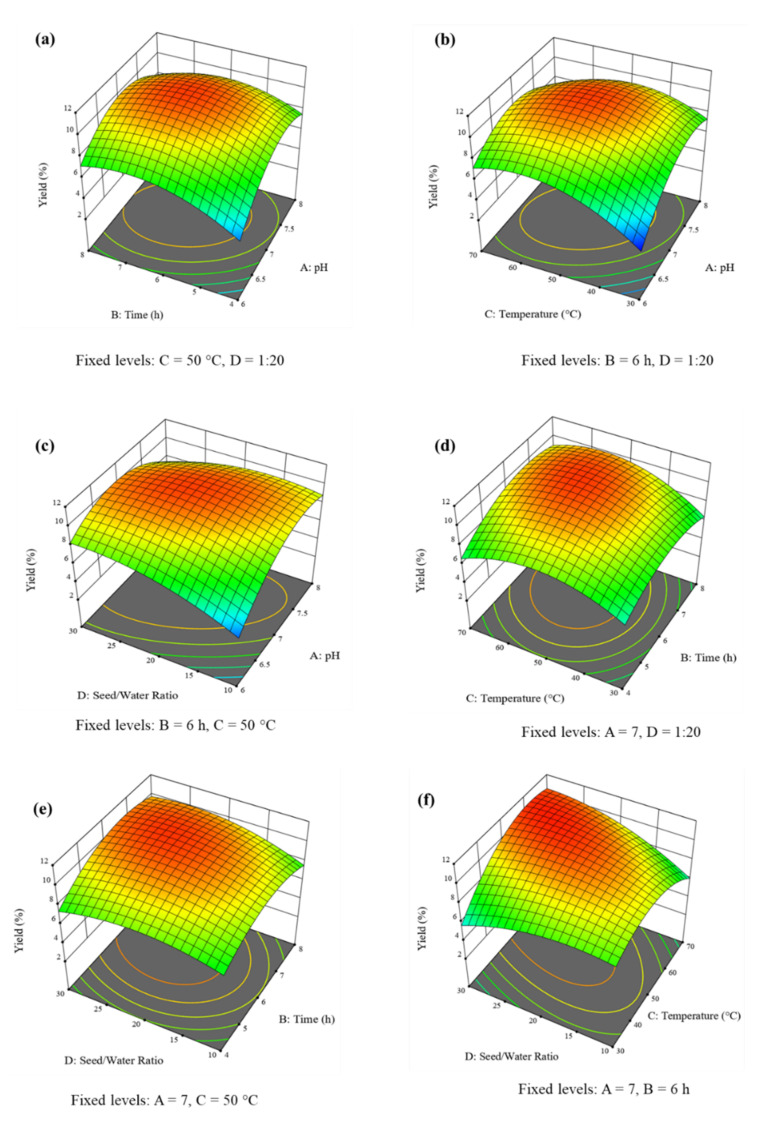
3D response surface plots pH vs. contact time (**a**), pH vs. extraction temperature (**b**), pH vs. seed/water ratio (**c**), contact time vs. extraction temperature (**d**), contact time vs. seed/water ratio (**e**), and extraction temperature vs. seed/water ratio (**f**), showing significant interaction effect on the yield of MPM (%) from MP seeds.

**Figure 3:**
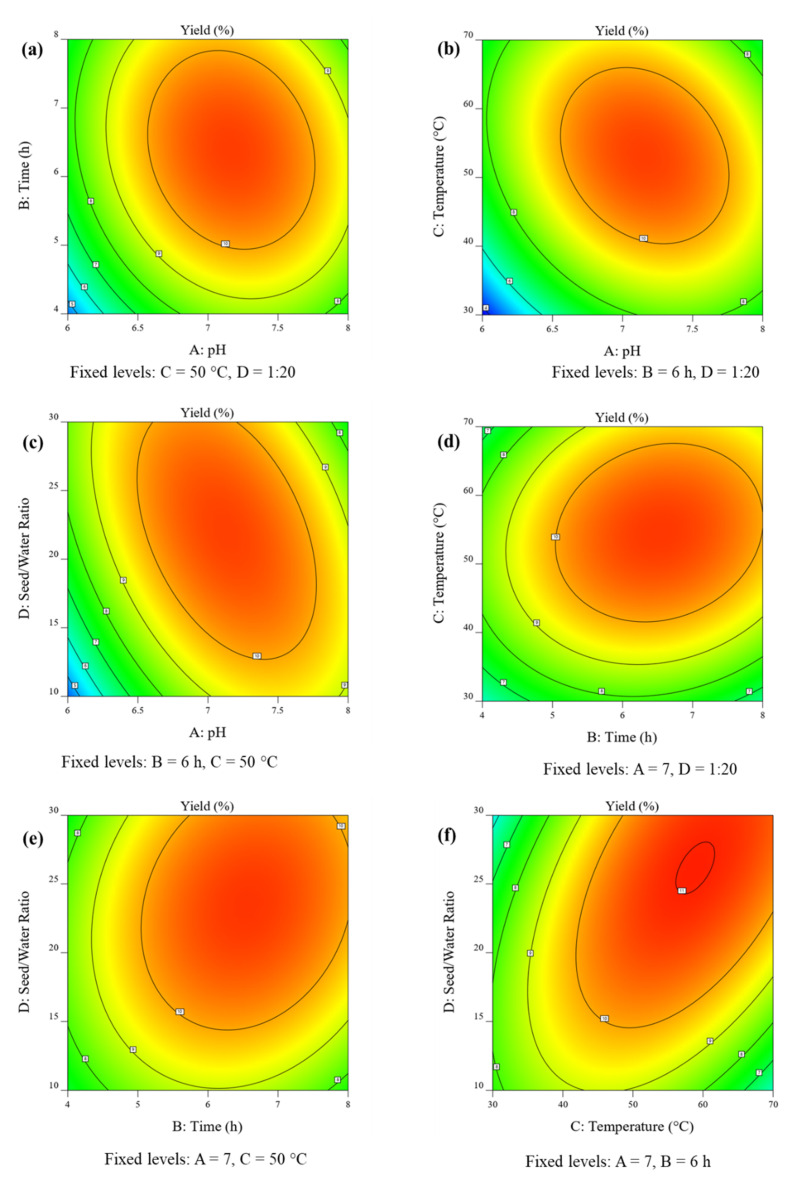
2D contour plots pH vs. contact time (**a**), pH vs. extraction temperature (**b**), pH vs. seed/water ratio (**c**), contact time vs. extraction temperature (**d**), contact time vs. seed/water ratio (**e**), and extraction temperature vs. seed/water ratio (**f**), showing significant interaction effect on the yield of MPM (%) from MP seeds.

The 3D response surface plot and 2D contour plots showing the combined effect of pH and extraction time on the yield of MPM at a constant temperature of 50 °C and seed/water ratio of 1:20 *w*/*v* are presented in Figure 2a and Figure 3a. It can be seen that, as the pH and seed/water ratio increased, the yield of MPM also increased and reached a maximum of 10.71% at pH 6.77 and contact time 7.26 h. However, after these threshold values, the yield of MPM decreased up to 7.93% at pH 7.95 and contact time 7.98 h.

The combined effect of pH and temperature on the yield of MPM is shown in Figure 2b and Figure 3b at a constant contact time of 6 h and seed/water ratio of 1:20 *w*/*v*. The yield of MPM increased from 3.71–10.77% by increasing pH from 6.0–7.21 and temperature from 30–54.77 °C. Beyond, these levels the MPM yield decreased to 7.36% at pH 7.96 and a temperature of 69.23 °C.

At constant contact time, 6 h, and extraction temperature of 50 °C, the finding of the effect of pH and seed/water ratio on the yield of MPM is shown in Figure 2c and Figure 3c. At low pH 6.0 and seed/water ratio 1:10.13 *w*/*v*, the yield of MPM was poor, i.e., 4.5%. However, by increasing pH and seed/water ratio, the MPM yield also increased significantly and reached a maximum of 10.75% at pH 7.1 and seed/water ratio 1:23.42 *w*/*v*. Later, the MPM yield decreased and achieved a minimum point of 7.57% at pH 7.98 and a seed/water ratio of 29.77 *w*/*v*.

The different extraction temperatures and contact also had significant effects on the yield of MPM. At an extraction temperature of 30 °C and contact time of 4 h, the yield of MPM was quite low, i.e., 6.0%. On increasing both of these parameters the yield of MPM increased to 10.83% at an extraction temperature of 55.47 °C and contact time of 6.47 h. Afterwards, the MPM yield decreased to 9.15% at an extraction temperature of 69.48 °C and a contact time of 7.98 h (Figure 2d or Figure 3d).

The quadratic effect of contact time and seed/water on the yield of MPM is graphically presented in Figure 2e and Figure 3e. The conditions of pH and extraction temperature were fixed at their optimum levels. It can be seen that as the contact time and seed/water ratio increased from 4.02–6.61 h and 1:10.09–1:22.74 *w*/*v*, respectively, the MPM yield also increased from 7.19–10.85%. The maximum yield of MPM, i.e., 10.85% was achieved at contact time 6.51 h and seed/water ratio 1:22.74 *w*/*v*. After these plateau regions, the MPM yield decreased to 9.88% at a contact time of 7.94 h and seed/water ratio 1:29.77 *w*/*v*.

The 3D response surface plot (Figure 2f) and 2D contour plot (Figure 3f) were recorded to study the dependency of the yield of MPM on extraction temperature and seed/water ratio at a fixed value of pH 7 and contact time of 6 h. The maximum yield of MPM, i.e., 10.99%, was obtained at an extraction temperature of 62.84 °C and seed/water ratio of 1:26.63 *w*/*v*. However, afterwards, a slight decrease in the MPM yield was observed to 10.61% at extraction temperature 69.58 °C and seed/water ratio 1:29.63 *w*/*v*.

In 3D response surface plots (Figure 2a–f), the areas that bulged out near 11% yield of MPM show the optimal regions of maximum yield. In 2D contour plots (Figure 3a–f), the regions demarcated by clear circular lines are showing the optimal regions for maximum yield of MPM.

### 3.5. Model Adequacy

**Figure 4:**
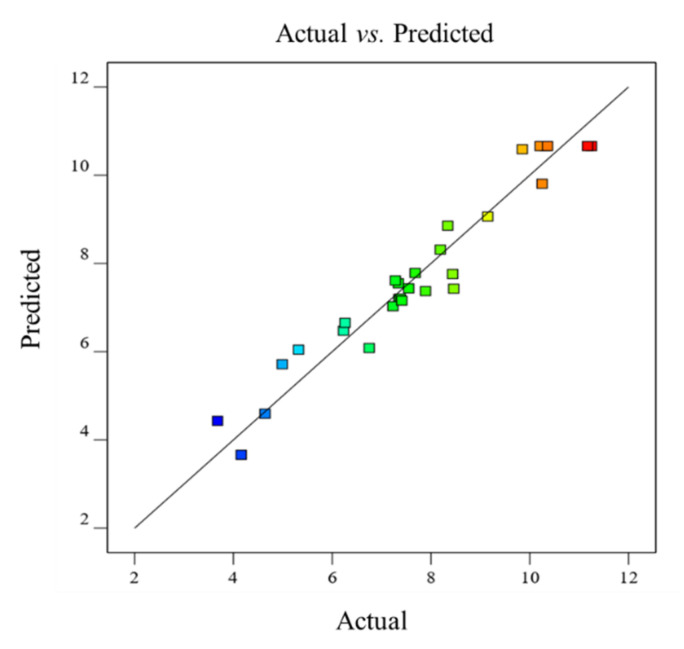
Comparison between actual vs. predicted yield of MPM obtained from MP seeds.

The RSM-BBD model adequacy was checked by plotting a graph between the actual (experimental) vs. predicted (theoretical) extraction yield of MPM (Figure 4; Table 2). The straight line in Figure 4 shows the actual yield of MPM whereas randomly displayed scattered points indicate the predicted yield. It is obvious that the values of the actual yield of MPM are very close to the predicted yield of MPM and are comparable. Hence, this good agreement between the actual and predicted yields illustrated that the second-order quadratic regression model satisfactorily described the yield of MPM by RSM-BBD.

### 3.6. Optimization of Extraction Yield and Checking of Model Desirability

**Figure 5:**
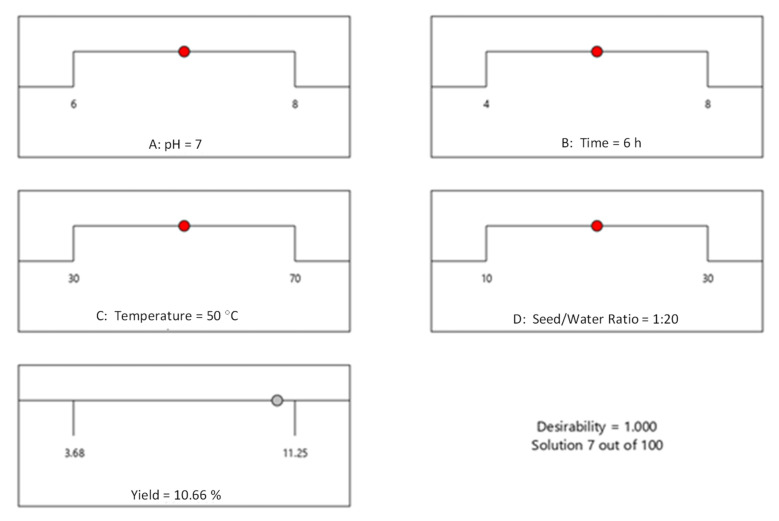
Desirability plot for the extraction optimization of MPM from MP seeds.

The graphical and numerical optimizations were recorded (Figure 2a–f and Figure 3a–f) and the maximum yield of MPM 10.66% was obtained at pH 7.0, contact time 6 h, extraction temperature 50 °C, and seed/water ratio 1:20 *w*/*v* by Design-Expert software. This optimized yield of MPM is close to experimental ones, i.e., 11.25% under similar extraction conditions (Table 2, run 5). The same is also evident from scattered plots between actual and predicted (Figure 4). Furthermore, the RSM-BBD model desirability for the extraction of MPM from MP seeds was found at 1.000 which also supported the ideal nature of the aforesaid conditions for the highest extraction yield of MPM from MP seeds according to RSM-BBD (Figure 5).

### 3.7. Comparison of Extraction Yield of MPM with Already Reported Mucilages

The extraction yield of MPM (11.25%) was found to be greater than mucilage from durian seeds (1.2%) [44], cress seed (6.46%) [45], chia seeds (6.96%) [3], kanocha seeds (7.35%) [46], and flax seeds (7.9%) [15]. Therefore, due to its high yield, MPM has the potential to become an important commercial gum.

### 3.8. pH-Responsive On–Off Switching Studies

**Figure 6:**
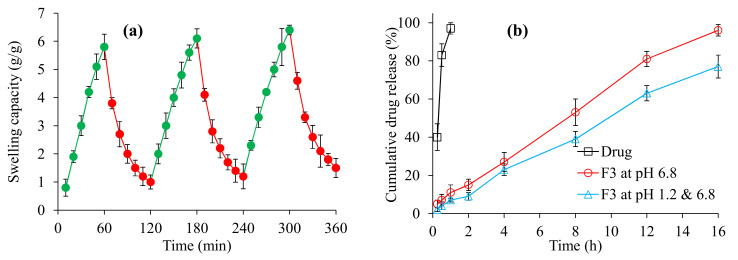
On–off switching (swelling and deswelling) studies of MPM-based oral tablet formulation F3 (**a**) and drug (itopride) release from MPM-based oral tablet formulation F3 at pH 6.8 and pH 1.2 (**b**).

The MPM-based oral tablet formulation F3 showed swelling at pH 7.4 and deswelling at pH 1.2 (Figure 6a). At pH 7.4, the carboxylic group (−COOH) present in the polymeric chains of MPM loses its protons and gets ionized to a carboxylate anion (COO^−^) which consequently offers anion-anion repulsions. Due to these repulsions, the adjacent polymeric chains repel each other and allow the swelling medium, i.e., the buffer of pH 7.4. to penetrate the polymeric matrix of MPM, which results in rapid swelling [47]. After record swelling at pH 7.4 for an hour, and upon shifting the tablet F3 to the deswelling medium, the COO^−^ ions accept protons from the deswelling medium, i.e., the buffer of pH 1.2, and become protonated to COOH [48,49,50]. Consequently, at pH 1.2, deswelling of F3 was observed. The on–off switching properties of F3 were observed up to three consecutive cycles and found to be reproducible.

### 3.9. In Vitro Release Study of Itopride

It was evaluated that MPM sustained the release of itopride for 16 h at pH 6.8. The net release of itopride was 96.0% after 16 h at pH 6.8 (Figure 6b). The release of itopride followed a zero-order kinetic pattern and super case-II transport mechanism by considering the values of *R*^2^ which is 0.9907, and *n* which is 0.915, respectively [25] (Table 4). The standard drug itopride was found completely dissolved at pH 6.8 within just 1.5 h (Figure 6a). Furthermore, in gastrointestinal tract mimicking conditions, a negligible amount of the itopride, i.e., <9.0% was released in the first 2 h at acidic pH, i.e., pH 1.2, whereas up to 77.0% of itopride was released after 16 h study at basic pH, i.e., pH 6.8. The less release of itopride at pH 1.2 is due to the inability of MPM to swell.

### 3.10. Applications and Future Research Perspectives

Extraction optimization of MPM from MP seeds via RSM clearly appeared to be a tool to reduce extraction time and economically enhance yield of mucilage. Hence, the findings of the presented research are valuable for future pharmacists and material chemists to develop new materials as Inactive Pharmaceutical Ingredients. Such materials, offering pH-sensitive swelling and deswelling responses are highly prosperous for the development of novel smart materials for intelligent drug delivery, due to their stimuli-responsive nature. Another perspective regarding mucilage, which is under discussion, is that it (MPM) could be a novel smart material for sustained/delayed/targeted drug delivery applications.

## 4. Conclusions

The RSM-BBD appeared to be an effective tool for the optimization of extraction conditions to get the highest yield of MPM from MP seeds. Results revealed that the pH of the extraction has a significant effect on the yield of MPM, followed by extraction temperature, contact time, and seeds/water ratio. A second-order quadratic model was obtained to predict the extraction yield of MPM. The RSM-BBD exhibited a high value of R^2^ (0.94255) and a non-significant lack of fit. The maximum yields of MPM 10.66% were obtained at pH 7, seed/water contact time of 6 h, extraction temperature of 50 °C, and seed/water ratio of 1:20 *w*/*v* by Design-Expert software, which is very close to experimental yield, i.e., 11.25%, under the same extraction conditions. Our results demonstrated that MPM is a material of superb choice for the development of a zero-order sustained release drug delivery system. The preliminary results of drug release studies appear promising and it is expected that MPM may also deliver different non-steroidal anti-inflammatory drugs and antibiotics to the colon after bypassing the stomach.

## Figures and Tables

**Table 1:** Composition of MPM-based oral tablet formulations in mg/tablet.

Constituents of the Tablets	F1	F2	F3
**MPM**	75	100	125
**Itopride**	100	100	100
**Microcrystalline cellulose**	120	95	70
**Magnesium stearate**	5	5	5
**Total weight**	300	300	300

**Table 2:** RSM-BBD experimental design and actual vs. predicted yield of MPM (%).

Run Nos.	Independent Variables				Yield (%)	
	pH A	Seed/Water Contact Time (h) B	Extraction Temperature (°C) C	Seed/Water Ratio (*w*/*v*) D	Actual Y	Predicted Z
**1**	7	6	30	10	8.44	7.76
**2**	6	6	30	20	4.16	3.66
**3**	8	6	70	20	7.23	7.03
**4**	7	4	30	20	5.32	6.05
**5**	7	6	50	20	11.25	10.66
**6**	7	4	70	20	6.26	6.65
**7**	7	8	70	20	9.15	9.06
**8**	7	6	70	10	6.75	6.08
**9**	8	6	50	10	8.34	8.85
**10**	7	8	50	30	10.25	9.81
**11**	7	8	30	20	6.23	6.47
**12**	8	6	50	30	7.55	7.44
**13**	8	6	30	20	7.89	7.37
**14**	7	8	50	10	7.28	7.62
**15**	8	8	50	20	7.68	7.78
**16**	7	4	50	10	7.41	7.16
**17**	6	6	50	30	8.19	8.31
**18**	7	4	50	30	8.46	7.43
**19**	6	6	70	20	7.38	7.20
**20**	7	6	70	30	9.85	10.59
**21**	8	4	50	20	7.34	7.55
**22**	7	6	30	30	4.99	5.72
**23**	7	6	50	20	10.36	10.66
**24**	7	6	50	20	11.17	10.66
**25**	6	4	50	20	4.64	4.59
**26**	7	6	50	20	10.31	10.66
**27**	6	8	50	20	7.35	7.20
**28**	6	6	50	10	3.68	4.43
**29**	7	6	50	20	10.21	10.66

**Table 3:** ANOVA of the experimental results of the RSM-BBD.

Source	Sum of Squares	DF ^a^	Mean	*F*-Value	*p*-Value ^b,c^
**Model**	110.10	14	7.86	16.39	<0.0001 **
**Linear**	9.42	1	9.42	19.62	0.0006 **
**A-pH**	6.04	1	6.04	12.57	0.0032 **
**B—Contact time (h)**	7.66	1	7.66	15.97	0.0013 **
**C—Temperature (°C)**	4.55	1	4.55	9.48	0.0082 **
**D—Seed/water ratio**	110.10	14	7.86	16.39	<0.0001 **
**Quadratic**					
**A^2^**	34.63	1	34.63	72.15	<0.0001 **
**B^2^**	15.95	1	15.95	33.23	<0.0001 **
**C^2^**	26.81	1	26.81	55.86	<0.0001 **
**D^2^**	7.71	1	7.71	16.07	0.0013 **
**Interaction**					
**AB**	1.40	1	1.40	2.93	ns
**AC**	3.76	1	3.76	7.84	0.0142 *
**AD**	7.02	1	7.02	14.63	0.0019 **
**BC**	0.9801	1	0.9801	2.04	ns
**BD**	0.9216	1	0.9216	1.92	ns
**CD**	10.73	1	10.73	22.35	0.0003 **
**Residual**	6.72	14	0.4799		
**Lack of Fit**	5.70	10	0.5696	2.23	ns
**Pure Error**	1.02	4	0.2558		
**Cor Total**	116.82	28			
***R*^2^ = 0.94255; Adjusted-*R*^2^ = 0.8850; Predicted-*R*^2^ = 0.7055; CV = 8.92%; Mean = 7.76; ADP = 14.0462**					

^a^ DF: Degree of freedom; ^b^ Significant (* *p* < 0.05); ^c^ Highly significant (** *p* < 0.01); ns: Not significant.

**Table 4:** Mathematical data of zero-order kinetics and Korsmeyer-Peppas model.

Formulation Code	Zero-Order Kinetic Model		Korsmeyer-Peppas Model		
	*R* ^2^	K_0_	*R* ^2^	K_KP_	*n*
**F3**	0.9907	6.758	0.9933	8.178	0.915
